# Wnt5a Deficiency Leads to Anomalies in Ureteric Tree Development, Tubular Epithelial Cell Organization and Basement Membrane Integrity Pointing to a Role in Kidney Collecting Duct Patterning

**DOI:** 10.1371/journal.pone.0147171

**Published:** 2016-01-21

**Authors:** Ilkka Pietilä, Renata Prunskaite-Hyyryläinen, Susanna Kaisto, Elisavet Tika, Albertien M. van Eerde, Antti M. Salo, Leonardo Garma, Ilkka Miinalainen, Wout F. Feitz, Ernie M. H. F. Bongers, André Juffer, Nine V. A. M. Knoers, Kirsten Y. Renkema, Johanna Myllyharju, Seppo J. Vainio

**Affiliations:** 1 Laboratory of Developmental Biology, Oulu Centre for Cell-Matrix Research, Biocenter Oulu and Infotech Oulu, and Faculty of Biochemistry and Molecular Medicine, University of Oulu, Oulu, Finland; 2 Department of Genetics, Center for Molecular Medicine, University Medical Center Utrecht, Utrecht, The Netherlands; 3 Oulu Centre for Cell-Matrix Research, Biocenter Oulu and Faculty of Biochemistry and Molecular Medicine, University of Oulu, Oulu, Finland; 4 Biocenter Oulu, Faculty of Biochemistry and Molecular Medicine, University of Oulu, Oulu, Finland; 5 Biocenter Oulu, University of Oulu, Oulu, Finland; 6 Department of Urology, Radboudumc Amalia Children’s Hospital, Radboud University Medical Center, Nijmegen, The Netherlands; 7 Department of Human Genetics, Radboud University Medical Center, Nijmegen, The Netherlands; National Cancer Institute, UNITED STATES

## Abstract

The Wnts can be considered as candidates for the Congenital Anomaly of Kidney and Urinary Tract, CAKUT diseases since they take part in the control of kidney organogenesis. Of them *Wnt5a* is expressed in ureteric bud (UB) and its deficiency leads to duplex collecting system (13/90) uni- or bilateral kidney agenesis (10/90), hypoplasia with altered pattern of ureteric tree organization (42/90) and lobularization defects with partly fused ureter trunks (25/90) unlike in controls. The UB had also notably less tips due to *Wnt5a* deficiency being at E15.5 306 and at E16.5 765 corresponding to 428 and 1022 in control (p<0.02; p<0.03) respectively. These changes due to *Wnt5a* knock out associated with anomalies in the ultrastructure of the UB daughter epithelial cells. The basement membrane (BM) was malformed so that the BM thickness increased from 46.3 nm to 71.2 nm (p<0.01) at E16.5 in the *Wnt5a* knock out when compared to control. Expression of a panel of BM components such as *laminin* and of *type IV collagen* was also reduced due to the *Wnt5a* knock out. The *P4ha1* gene that encodes a catalytic subunit of collagen prolyl 4-hydroxylase I (C-P4H-I) in collagen synthesis expression and the overall C-P4H enzyme activity were elevated by around 26% due to impairment in *Wnt5a* function from control. The compound *Wnt5a*^+/-^;*P4ha1*^+/-^ embryos demonstrated *Wnt5a*^-/-^ related defects, for example local hyperplasia in the UB tree. A R260H WNT5A variant was identified from renal human disease cohort. Functional studies of the consequence of the corresponding mouse variant in comparison to normal ligand reduced Wnt5a-signalling *in vitro*. Together Wnt5a has a novel function in kidney organogenesis by contributing to patterning of UB derived collecting duct development contributing putatively to congenital disease.

## Introduction

Congenital anomaly of the kidney and urinary tract (CAKUT) is a collective definition for a spectrum of structural kidney malformations that have their origin *in utero* being a frequent malformation type [1]. Due to this and the fact that CAKUT can progress to the end-stage renal disease (ESRD) it is a considerable economic burden [2]. Better understanding of kidney organogenesis should identify novel CAKUT candidates [3].

Wealth of factors that control kidney development have been identified during recent years [4,5]. Initiation of organogenesis involves GDNF and its Ret receptor in the prospective collecting duct derived from the ureteric bud (UB) to advance UB arborisation in concert with FGF, BMP and Wnt growth factors (GFs) [6,7]. The Wnt family members are involved, particularly in nephrogenesis [8–14]. Recently Wnt5a was implicated as a signal in kidney organogenesis [15–17] and based on the data in a cell model polarized Wnt5a secretion may advance lumen formation in tubulogenesis [18] with integrins [19].

Integrins and cell surface proteoglycans (PGs) and their extracellular matrix (ECM) ligands contribute to kidney organogenesis as well [20,21]. The ECM accumulates GFs such as the Wnts to localise their signalling and availability [22]. In the context of organogenesis GFs encounter a specialized ECM lamina, the basement membrane (BM) that is considered to be involved in the reciprocal epithelial and mesenchyme tissue interactions coordinating organogenesis. For example, the ECM component type XVIII collagen binds Wnts and antagonises their signalling [23]. How the concerted actions between BM, ECM and GFs regulate morphogenesis is still unclear.

Wnt5a appears to serve as a signal in coordination of UB development. It promotes prospective collective duct epithelial cell organization and the associated basement membrane formation such that type IV collagen and laminin expression is reduced in the absence of Wnt5a signalling. A R260H human WNT5A variant was identified in a human CAKUT cohort. This variant had reduced signalling in a model cell experiments. Thus Wnt5a advances prospective collecting duct development during kidney organogenesis at least in part via promotion basement membrane and the organization of the overlying epithelial cells.

## Materials and Methods

### Mouse models

Generation and genotyping protocols of the *Wnt4*, *Wnt5a* and the *P4ha1* knockout mice have been described previously [9,24,25]. The *Wnt4*^*-/-*^;*Wnt5a*^*-/-*^ was generated by crossing *Wnt4*^*+/-*^ and *Wnt5a*^*+/-*^ mice. The embryos were considered to be E0.5 at noon of the day of the appearance of the vaginal plug. The pregnant females were sacrificed using CO_2_ and cervical dislocation and the embryos were decapitated before sample collection to alleviate suffering. The animal care and experimental procedures were conducted in accordance with the Finnish national legislation for the use of laboratory animals, the European Convention for the protection of vertebrate animals used for experimental and other scientific purpose (ETS 123), and EU Directive 86/609/EEC.

### Histology and immunohistochemistry

For histology the kidneys were prepared from mouse embryos at E10.5-E17.5 in ice-cold PBS, pH 7.3, and subjected immediately to 4% paraformaldehyde (PFA). After washes and dehydration the tissues were embedded in paraffin and sectioned serially for histology and immunological staining. Hematoxylin/eosin and periodic acid-Schiff staining (PAS) were used for the tissue sections. Primary antibodies against collagen IV (Millipore, AB756P, MA USA), laminin (Sigma-Aldrich, L9393, USA), Troma-1 (Hybridoma Bank, Iowa USA), caspase-3 (Cell Signalling, USA) and Ki-67 (DakoCytomation, M7249) were used. The Alexafluor 488, 594 or 546 conjugated antibodies (Molecular Probes, Oregon, USA) were used as secondary antibodies. The sections were inspected with the microscopes Olympus SZX12 (Japan) and Olympus FluoView FV1000 (Japan) that were attached to a CCD camera to document the sections. Photoshop CS5, F10-ASW 3.0 Viewer and Imaris were applied to process the images.

### *In situ* hybridization

Section and whole mount *in situ* hybridization was performed as described previously [13,26]. The prepared *Wnt5a*^-/-^, *Wnt5a*^+/-^ and wild-type embryonic kidneys were fixed in 4% PFA and subjected besides sectioning to whole mount *in situ* hybridization. For this purpose hybridization of the probes and the washes afterwards were performed with the aid of the Insitupro (Intavis AG Bioanalytical Instruments, Koeln Germany) or BioLane^TM^ HTI (Hölle & Hüttner AG, Germany) robots. The RNA probes to localise the *Wnt5a* and *Wnt11* transcripts were generated from linearized plasmids that were obtained as gifts from Prof. A.P. McMahon (Univ. Southern California, USA). The stained samples were inspected with an Olympus SZX12 (Japan) microscope, photographed with the Olympus CCD camera and processed with Photoshop CS5.

### Optical projection tomography (OPT)

The optical projection tomography (OPT) technology was applied to obtain virtual, digital three dimensional (3D) images of the whole kidney and the main tissue compartments, the ureteric tree. The dissected kidneys (E15.5–16.5) were stained as whole mount with the Troma-1 antibody (Hybridoma bank, Iowa USA) [27,28]. The kidneys were mounted to 1% low melting point agar and dehydrated overnight (o/n) in methanol at RT. The samples were cleared o/n at 4°C with benzyl alcohol and benzylbenzoate (1:2) and imaged with the OPT Scanner 3001M (Bioptonics Microscopy, UK). The Imaris software (Bitplane, Zurich Switzerland) was used to analyze the OPT data. The branching morphology was assessed by filament tracing plug in and the branching angles were measured by Branching angle B function. It measures the angle between the root branch and extending neighbouring branching points.

### Generation and analysis of the R260H Wnt5a variant

An R260H point mutation was made to the mouse *Wnt5a* cDNA sequence with a QuikChange II XL Site-Directed Mutagenesis Kit (Agilent, CA USA). The putative impact of this point mutation was analysed by studying the capacity of the wild-type and the R260H Wnt5a to inhibit the Wnt3a induced TOP Flash reporter assay as described previously [29].

### Quantitative real-time PCR (qRT-PCR)

The kidneys were dissected and snap frozen in liquid nitrogen to determine mRNA expression by qRT-PCR. Possible Wnt5a induced changes in gene expression were analysed also in an embryonic kidney-derived MK3 cell line. For this purpose, the cells were transduced with a virus expressing Wnt5a-GFP or GFP only. The total RNA was purified from wild-type, *Wnt5a*^-/-^ kidneys and MK3cells with the RNeasy Mini Kit (Qiagen, Germany) and the cDNA was synthesized with the First Strand cDNA Synthesis Kit (Fermentas). The primers used to amplify the selected gene sequences are Col IV a1 TCCGGGAGAGATTGGTTTCC, CTGGCCTATAAGCCCTGGT; Col IV a2 GGACCCAAGGGACAACCAG, CCCAACAAGTGTGATGTCAGAT; Col IV a3 CAAAGGCATCAGGGGAATAACT, ACCCTTAGATCCGTTGCATCC; Ln a2 TCCCAAGCGCATCAACAGAG, CAGTACATCTCGGGTCCTTTTTC; Ln b1 GAAAGGAAGACCCGAAGAAAAGA, CCATAGGGCTAGGACACCAAA; LN b2 GAACTTCGCTTGGGCCTACTT, GGTGGCTGGATAGCAGCTT; Ln c2 TCGTATCAGCACAGTCTCCG, GCAACCTTCTGGCTAATAGAGG; Ln c3 TTGCAGGGCAGACACTCGTT, GTCCTCCAGTCTTACTACTACG; GAPDH AGAACATCATCCCTGCATCC, CAGTGAGCTTCCCGTTCAG. The qRT-PCR program consisted of 40 cycles at 95°C for 30 sec and at 60°C for 1 min in an MX3005 thermocycler (Stratagene, La Jolla, Ca, USA). The qPCR experiments were performed as triplicates and the data was normalized to GAPDH expression using the ΔΔCT method.

### Prolyl 4-hydroxylase activity assay

The kidneys were dissected at E16.5 and homogenized in a solution containing 0.1M NaCl, 0.1M glycine, 0.1% Triton X-100, 10 mM Tris, pH 7.8, with Complete EDTA-free protease inhibitors (Roche, Basel Switzerland), and centrifuged at 10,000 × g for 20 min. The protein concentration in the soluble fraction of the cell lysates was determined by the Bio-Rad Protein assay (Bio-Rad, Hercules, CA, USA). Prolyl 4-hydroxylase activity was measured by determining the amount of 4-hydroxy-[^14^C]proline generated in [^14^C]proline-labeled nonhydroxylated chicken type I procollagen chains [30],[31].

### Electron microscopic analysis

Freshly dissected kidneys were prepared from E16.5 *Wnt5a* knockout and control littermate mouse embryos and subjected to 1% glutaraldehyde, 4% PFA in 0.1M PBS for electron microscopic (EM) sample preparation. The samples were processed and analysed by the EM core facility laboratory of Biocenter Oulu (Oulu, Finland). The images were captured with the Tecnai G2 Spirit 120 kV transmission electron microscope.

### Human derived DNA samples

Written informed consent was obtained from all the patients and parents. If the patients involved children, the parents or legal guardians were asked to give informed consent on their behalf. Children 12–17 years of age signed the consent form together with their parents. The Committee on Research involving Human Subjects approved the study protocol under agreement number CMO-048/2006.

Duplex collecting system patients (n = 96) were recruited at the University Medical Center Utrecht (Utrecht, The Netherlands) and the Institutional Review Board of the UMC Utrecht approved this. DNA from renal agenesis patients (n = 15) was available from the AGORA (Aetiological research into Genetic and Occupational/environmental risk factors for Anomalies in children) bio bank project (Radboud university medical center, Nijmegen, The Netherlands). This part of the study was approved by the Arnhem-Nijmegen Regional Committee on Research Involving Human Subjects. From the Maastricht University Medical Centre (Maastricht, The Netherlands) we obtained 14 DNA samples of patients that were diagnosed with Alport syndrome and were negative for *COL4A5*, *COL4A3*, and *COL4A4* mutations. Informed consent was obtained from all the patients for the DNA analyses in this study. Peripheral blood samples were obtained and the DNA was isolated from lymphocytes using standard procedures. The patients and their diagnosis are summarized in Table 1.

**Table 1:** Description of the phenotypes in patient with CAKUT.

Phenotype	Unilateral/bilateral	n
Duplex collecting system	Unilateral	78
	Bilateral	18
Renal Agenesis	Unilateral	12
	Bilateral	3
Horseshoe kidney		4
Alport syndrome		14
**Total**	** **	**129**

### Sequencing of the *WNT5A* gene in CAKUT patients

The coding region and the intron-exon boundaries of the human *WNT5A* gene (NM_003392) were analysed by Sanger sequencing. The applied primers are; Exon 1 AGTGATCTCCTGGGACACTG, TCAGCTCCGGTTCACTG; Exon 2 CCATTCCCTAGGAGCTGAAG, AATCAGATTTCCTGGTGAGG; Exon 3 TCATCAGGTGTAGGGACAGG, TCATGAGGACAAGCAGGAG; Exon 4 ATAGCAAAGGAGTGGCAGAG, CCACCATTCCCTACCTTG; Exon 5a GTGCACTTCTTGCACTTGAC, GGAGAAGGTCGAGGAGAAC; Exon 5b CAGAGTTCTTAGATGGTAACAGG, CATGTAGCCTGAAGACATGC.

*WNT5A* gene mutation analysis was performed in 129 patients. The in-house and the online available databases dbSNP and Exome Variant Server of the NHLBI Exome Sequencing Project were used to determine whether *WNT5A* variants had been detected previously. DNA samples that were derived from 189 healthy Dutch blood donors served as controls and were screened for the presence of the identified *WNT5A* variant.

### *In silico* modelling of the putative structural effect of the identified WNT5A variant

Possible consequences of the R260H mutation on the structure and dynamics of the WNT5A protein were studied by conducting 20 nanoseconds molecular dynamics (20ns MD) simulations of the wild-type WNT5A and the mutant R260H WNT5A. The I-TASSER server [32] was used to generate a model of the human WNT5A protein structure. WNT5A appeared to have 41.5% sequence similarity to Wnt8f for which a crystal structure is available [33] (PDB:4F0A). The Wnt8f structure was found to be the best available template and it was used to produce models of the WT WNT5A and the R260H mutant. Every model was used to perform an independent MD-simulation. The simulation boxes were cubes of 987.5nm^3^. The putative computer-generated WNT5A protein models were placed in the centre of the cube. The models were solvated with 31100 SPC water molecules.

To reach an ion concentration of 0.15mM and neutralize the electrostatic charges on the WT protein, 89 water molecules were replaced by Na^+^ ions and 99 by Cl^-^. Similarly, 73 Na^+^ and 82 Cl^-^ ions were used for the mutant. Both systems were equilibrated through an energy minimization run and a ten ps position restrained simulation. The obtained coordinates were used to perform in the full 20 nanosecond simulations.

To account for the presence of ligands in the template structure and to focus our observations on the putative effects of the noted R260H mutation, position restrains of 1000 kJ mol-1 nm-1 were applied to the residues 30 to 49 and 222 to 380 in the WNT5A protein. All the simulations and their required setup were performed using the GROMACS software package and the GROMOS 53A6 [34] force field. The simulation conditions were set to a 300K temperature and a pressure of one bar with the aid of the Berendsen thermostats and barostats. The T-Test gave a P value of 0 (up to 20 decimal places).

## Results

### Failure in *Wnt5a* signalling influences ureteric tree development leading anomalies characterized in CAKUT

**Fig 1:**
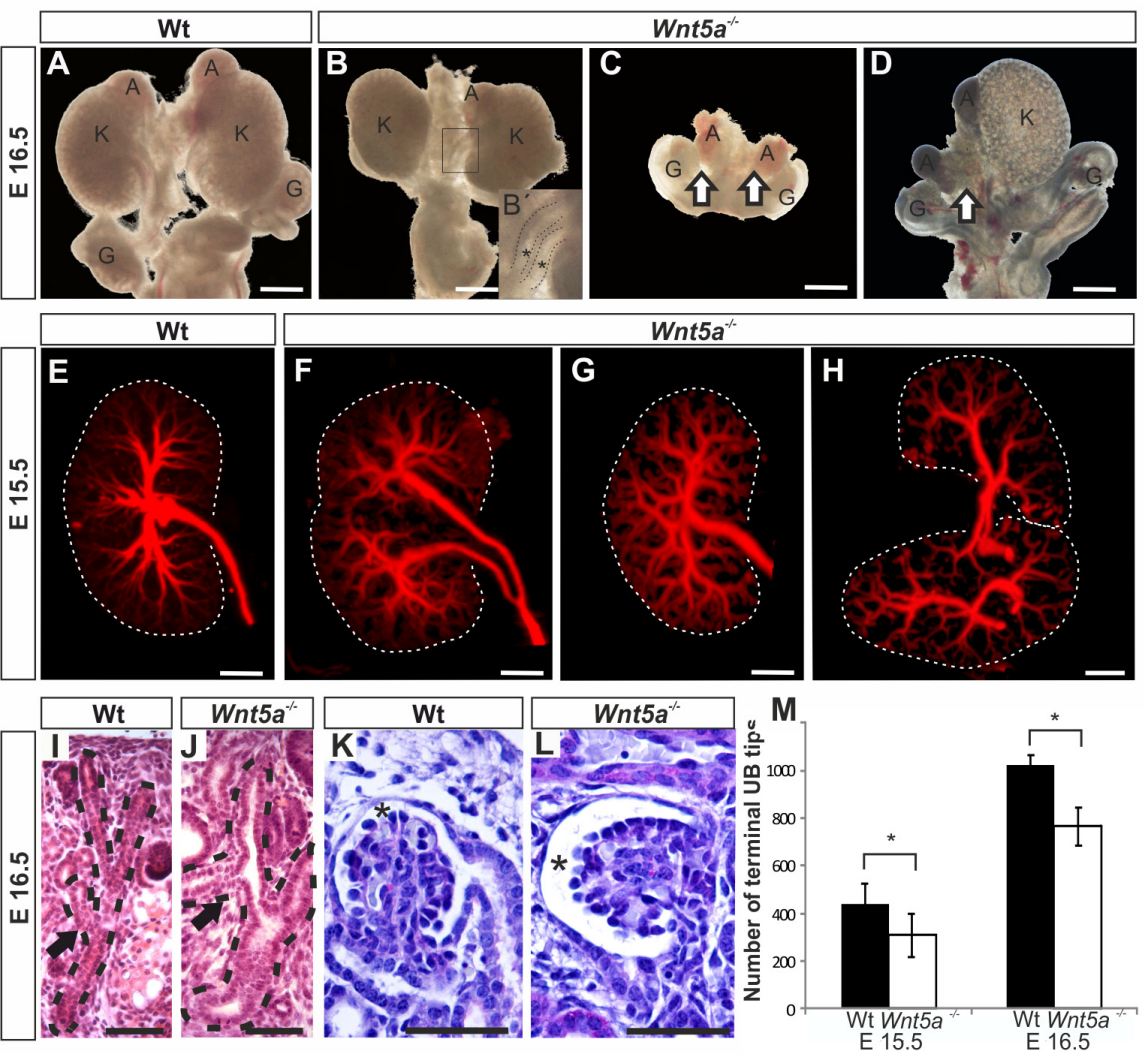
*Wnt5a* deficiency leads to severe metanephric kidney anomalies. Embryonic urogenital systems (UGS) (A-D) and their kidneys (E-L) were dissected at E15.5 and E16.5. The UGS were inspected either as unstained (A-D) or their kidneys we subjected to Troma-1 antibody staining as whole mount and analysed with the optical projection tomography (OPT) to identify the pattern of the ureteric bud (UB) tree and the terminal UB derived tree tip counts (M) or sectioned (I-L). A) A normal urogenital system. The kidney (K), the gonad (G) and the adrenal gland (A) are marked. The *Wnt5a* deficiency leads to three categories of phenotypes; B) a kidney with duplex UB highlighted in the boxed image (the stars in B´), kidney hypoplasia (compare B with A), bilateral (C) or unilateral (D) renal failure. The OPT reveals variation noted in the pattern of UB tree development in the *Wnt5a* deficient embryonic kidneys when compared to control (compare F—H with E). The altered UB tree pattern can be depicted in the sections of the *Wnt5a* deficient kidneys when compared to control (compare J with I, dotted line, arrows). *Wnt5a* deficiency enlarges also the Bowman´s capsule lumen (asterisk) from control one (compare L with K, stars). Counting of the UB terminal tips from the OPT data indicates reduction in their number due to *Wnt5a* deficiency from controls M). Data in M are shown as means ± SD, *n* = 4–5 kidneys/group. Scale bars, A-D 400 μm, E-H 200 μm and I-L 50 μm. * *P* <0.05.

*Wnt5a* is expressed in the intermediate mesoderm cells prior to metanephros development [16] and becomes confined later to the Ret+ epithelial UB cells [15]. Renal roles of the *Wnt5a* were studied via the deficient mouse model [24]. The analysis of urogenital systems of normal and the deficient embryonic kidneys at E15.5 and E16.5 allowed classification of the noted phenotypes into four categories: presence of duplex collecting system (13/90) (Fig 1, compare B, F with A, E, arrows, S1 Movie), uni- or bilateral kidney agenesis (10/90) (Fig 1C and 1D, arrows), kidney hypoplasia associated with altered pattern of ureteric tree organization (42/90) (Fig 1, compare G with E) and lobularization defects with partly fused ureter trunks (25/90) not noted in controls (Fig 1, compare H with E).

Histology at E15.5 suggested that the *Wnt5a* deficiency would change spatial UB organization and the diameter of the UB branches that appeared visually larger than controls (Fig 1, compare J with I, shaded regions, arrows). The nephrons still formed in the absence of *Wnt5a* function but the Bowman’s capsule became dilated later (Fig 1, compare L with K, asterisk).

We used the optical projection tomography (OPT) that offers quantitation of the morphological anomalies in the *Wnt5a* deficient kidneys. The OPT data revealed that at E15.5 the terminal branching tips of the Wnt5a ^-/-^ mouse ureter tree were decreased from WT controls 428 tips to 306 tips (p<0.02) and E16.5 from 1022 to 765 (p<0.03) respectively. (Fig 1M). In agreement with the histological findings (Fig 1E–1H), the OPT indicated an overall increase in the UB tree branching angles in *Wnt5a* deficient kidney in comparison to control, being 38.10 and 43.14 degrees respectively (p<0.05). Together the findings point towards a possibility that Wnt5a appears to take part in the control of UB derived collecting duct tree development.

### Changes in polarization of the *Wnt5a* deficient epithelial ureteric tree cells correlates with severely deregulated basement membrane organization

One of the typical manifestations of epithelial UB tree cell polarity is the associated polarized secretion of the BM components between the kidney assembling UB and the MM tissues by the UB cells. The noted defects in the overall altered UB tissue pattern due to *Wnt5a* deficiency share resemblance to defects in the *laminin γ1* knock out [35]. Thus we considered that the Wnt5a signal may coordinate UB development via involvement of the BM, its components such as laminins. This was addressed by dissecting embryonic kidneys at E11.5, E14.5 and E16.5 and by subjecting them to EM inspection.

**Fig 2:**
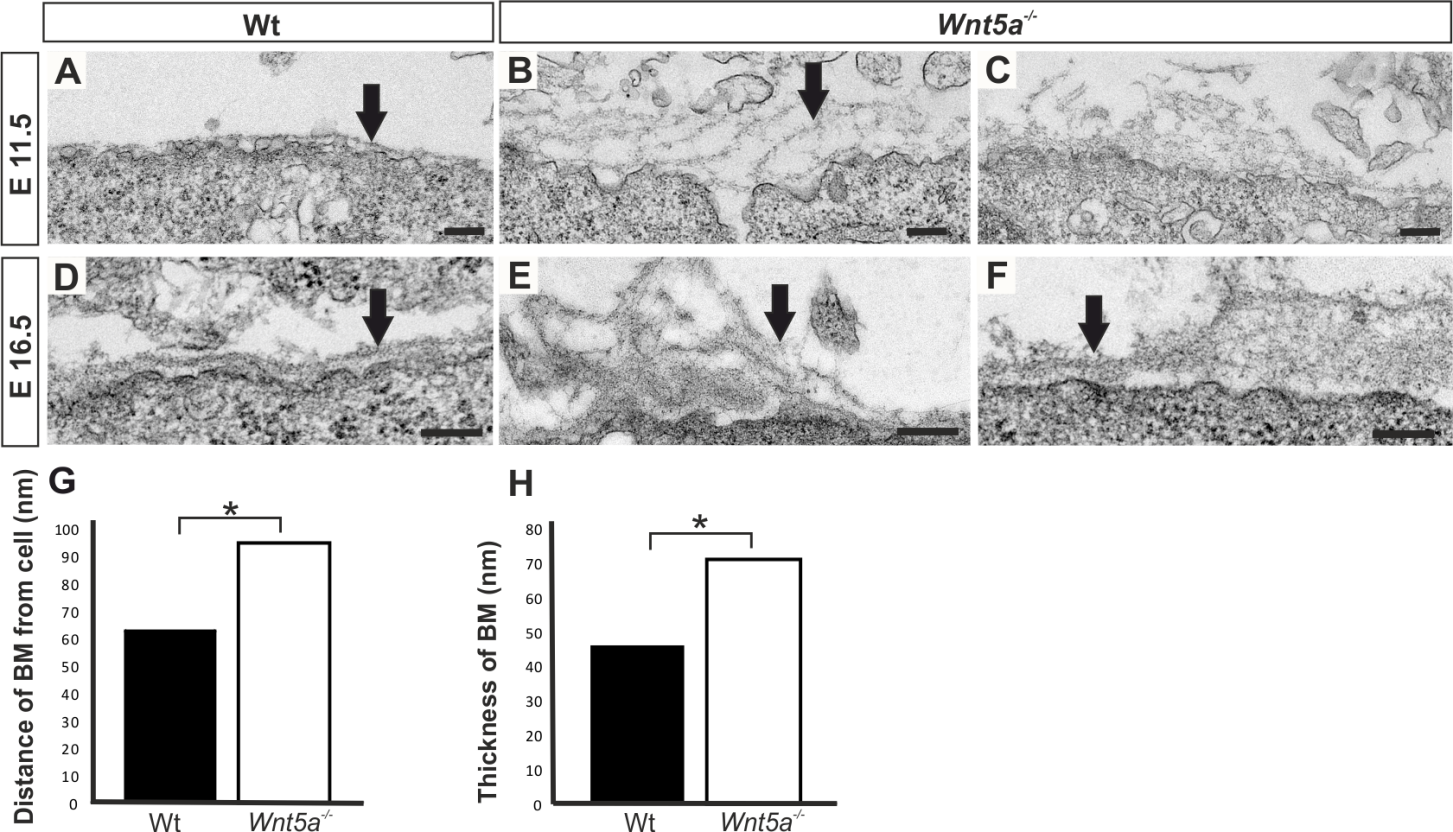
*Wnt5a* deficiency associates to compromised basement membrane integrity and integrin *Itga1* expression. Wild-type (Wt) and *Wnt5a*^-/-^ kidneys at E11.5 and E16.5 were prepared and subjected to electron microscopic (EM) inspection (A-F). The noted phenotypes were quantified by morphometric (G-H). The basement membrane (BM) that forms normally an extracellular matrix (ECM) sheet between ureteric bud derived epithelial tree and adjacent mesenchyme (A, D) becomes severely compromised in the *Wnt5a* deficient embryonic kidney already at the initiation of organogenesis at E11.5 (compare B, C with A, arrows) and is prominent also at E16.5 (compare E, F with D, arrows). Please note that the BM of the *Wnt5a* deficient kidney loses it integrity and the BM is more extended when compared to control BM (compare B, C, E, F with A, D, arrows). G, H) Quantitation of the BM (A-F) in depicting that the width of the BM and the distance of the BM from the cell membrane is increased in the absence of *Wnt5a* function when compared to control. Data are presented as means ± SD. *p < 0.05, n = 4 mice/group. Scale bars, A-F 200 nm.

At E11.5 when kidney organogenesis has initiated and a primitive BM is distinguishable between UB and MM (Fig 2A, arrow) the BM integrity of the *Wnt5a* deficient UB was compromised. The BM formed had become detached and fragmented in many locations. The BM had also formed folds that were not detected in wild-type (Fig 2, compare B, C with A, arrows). The noted *Wnt5a* deficiency related phenotypes at E11.5 were noticeable at E 14.5 and E16.5 (Fig 2, compare E, F with D, arrows).

Distance of ECM edge from cell surface and overall BM thickness at E16.5 were higher in the *Wnt5a* deficient samples than in control. At E14.5 the BM distance from plasma membrane was increased from WT 57.4nm to 90.4nm in *Wnt5a* ^*-/-*^ and the thickness of BM was increased from WT being 40.0 and 58.9nm in *Wnt5a*^*-/-*^. At E16.5 the BM had increased its width to 71.2nm while in WT controls the BM width was 46.3 nm (p<0.005) and the BM distance from the plasma membrane was 62.9nm in WT while in *Wnt5a*^*-/-*^ it was increased to 95.1nm (p<0.01) (Fig 2G and 2H and data not shown).

To further substantiate the point of Wnt5a serving as a cell polarity cue to promote concurrently the polarized ECM secretion we localized type IV Collagen and Pan-Laminin expression in sections. In comparison to WT control, the expression of both type IV Collagen and Pan-Laminin were reduced in *Wnt5a* deficient samples (S1 Fig, compare B, D with A, C, arrows). Similarly, *Lama1*, *Lamb1*, *Lamc2 and Lamc3*, *Col4a1*, *Col4a2* and *Col4a3* except *Lamb2* were reduced due to the *Wnt5a* deficiency as judged by qRT-PCR (S1E Fig) and was the case with Ln-111 depicted with immunoblotting (S1G Fig). Taken together the evidence points towards a conclusion that Wnt5a contributes to ureteric tree development via maintenance of the epithelial cell BM. One indicative output of this is the promotion of efficient polarized secretion of the ECM components between the UB and MM tissues.

### Enhanced collagen biosynthesis in the *Wnt5a* deficient kidney and the phenotypes generated by heterozygosity of the *Wnt5a/Collagen Prolyl 4-Hydroxylase* genes

**Fig 3:**
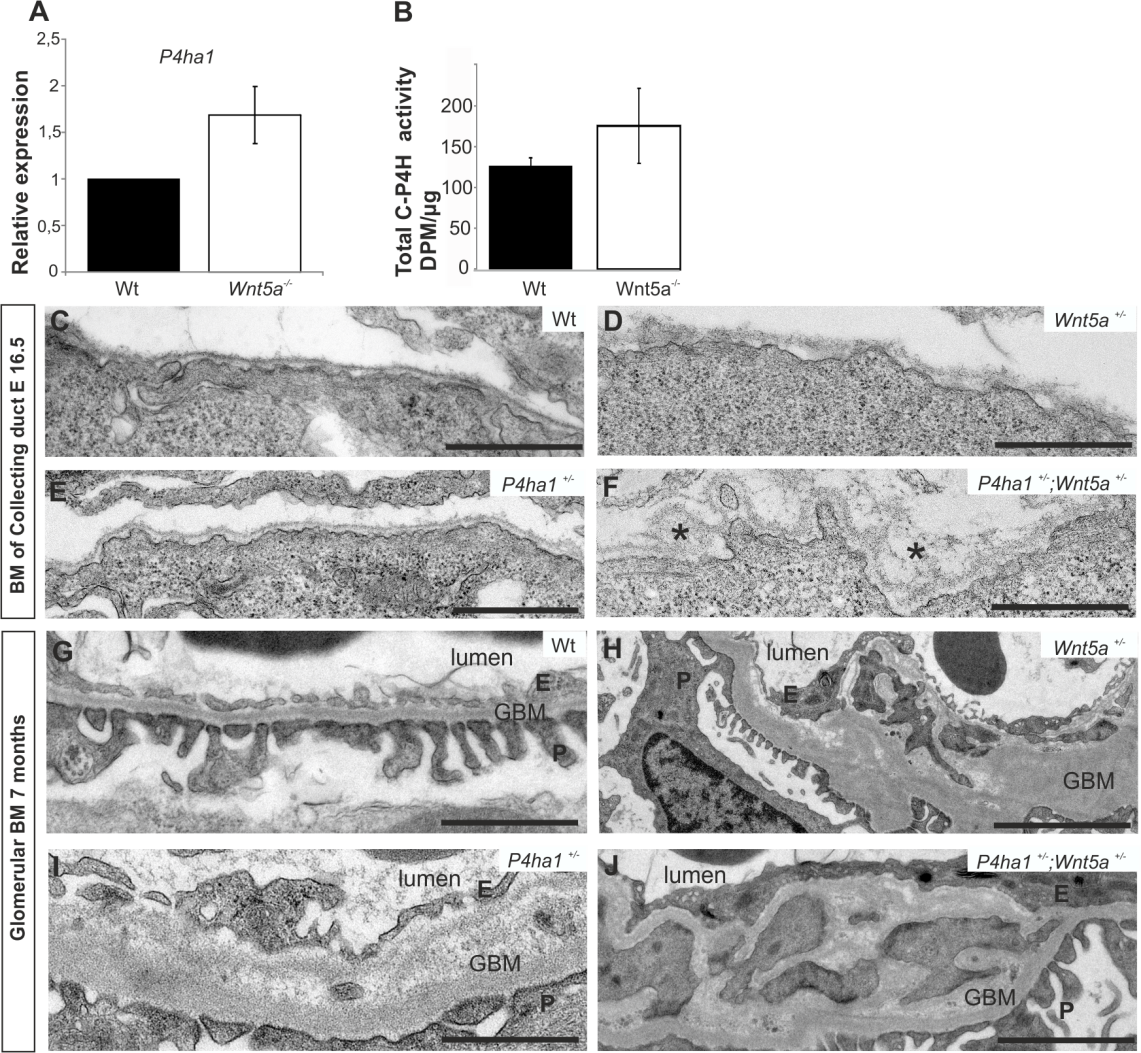
*Wnt5a* deficiency increases the collagen prolyl 4-hydroxylase I (P4ha1) expression and the compound +/- is characterized by severe kidney filter anomaly. The kidneys were prepared and subjected to *collagen prolyl 4-hydroxylase I (P4ha1)* gene expression (A), total Collagen P4H (C-P4H) enzyme activity assays (B) or electron microscopic inspection (C-J) at E16.5 or in the adult. *Wnt5a* deficiency has increased the *P4ha1* gene expression (A) and C-P4H enzyme activity (B) when compared to control. The ureteric bud derived collecting duct basement membrane (BM) remains normal at E16.5 in the *Wnt5a+/-* and *P4ha1+/-* (compare D, E with C) but the BM is malformed in the *P4ha1+/-; Wnt5a+/-* at E16.5 (compare F with C, D, E). However, the kidney filter, the glomerular BM and the foot processes are compromised in the kidney of the *P4ha1+/-* and the *Wnt5+/-* mice at the age of seven months (compare H, I with G) and this is even more severe in the kidneys derived from the *P4ha1+/-; Wnt5a+/-* compound heterozygous mice (compare J with G, H, I). GBM; glomerular BM, P, podocyte, E, endothelial cell. The data in A and B are shown as means ± SD, n = 4. Scale bars, C-J 1μm.

We found that *Wnt5a* deficiency influences spatial organization of the BM and decreases a panel of BM component expression. Based on this we speculated that the collagen synthesis machinery may play a role in the developmental chain regulated by *Wnt5a* in developing UB normally. Indeed *P4ha1* gene expression that encodes a catalytic subunit of collagen prolyl 4-hydroxylase I (C-P4H-I) in collagen synthesis and the overall C-P4H enzyme activity were elevated, the enzyme being around 26% higher by impairment in *Wnt5a* than in control (Fig 3A and 3B).

We assayed the putative consequences of the elevated C-P4H-I amount by reducing it *in vivo* via crossing the *Wnt5a*^*+/-*^; *P4ha1*^*+/-*^ mice together. The *P4ha1* null embryos fail to develop due to impairment to assemble type IV collagen integrated BM [36]. While the BM remained intact in the *Wnt5a*^*+/-*^ and *P4ha1*^*+/-*^ embryonic kidneys at E16.5 as expected (Fig 3, compare D, E with C) that of the compound *Wnt5a*^+/-^;*P4ha1*^+/-^ demonstrated severe defects at E16.5 as in the *Wnt5a*^-/-^ ones (Fig 3, compare F, asterisks, with C-E and with Fig 2E and 2F). PAS staining of the E16.5 *Wnt5a*^+/-^;*P4ha1*^+/-^ kidneys revealed local hyperplasia in the UB tree as in the *Wnt5a* deficient samples unlike in control (data not shown). Besides this the glomerular kidney filter was malformed even in the *P4ha1*^*+/-*^ and *Wnt5a*^*+/-*^ adult mice invaded by cells with mesangial cell characteristics (Fig 3, compare H, I with G). The anomalies in the glomerulus were notable worse in the *Wnt5a*^+/-^;*P4ha1*^+/-^ adult mice (Fig 3G–3J) being in line with a severe glomerulosclerosis condition. Thus reduction of the *P4ha1* mediated compensation in the absence of *Wnt5a* function aggravates the renal disease phenotype.

### Mutation in *WNT5A* may serve as a susceptibility factor for CAKUT in humans

The findings here in implicate that Wnt5a is a critical murine signal for kidney UB tree development and provided a candidate for human CAKUT when expression would have been compromised *in utero*. We sequenced the coding region and the intron-exon boundaries of human *WNT5A* (NM_003392) in a cohort of 129 CAKUT and Alport syndrome patients with either a duplex collecting system, renal agenesis, horseshoe kidney or Alport syndrome with unknown cause (Table 1).

The screen depicted a novel heterozygous *c*.*779G>A WNT5A* variant in a patient with a unilateral duplex collecting system (S2A Fig). The father of the patient had the same variant. However, the ultrasound analysis did not reveal a duplex collecting system in the father. It should be considered that the current analysis capacity may not be sensitive enough to determine the putative decrease in the ureteric branching in the father. The mother was diagnosed to have CAKUT however.

It is noteworthy that the here in identified variant leads to a p.R260H transition in a conserved WNT5A region (S2B Fig). The PhyloP conservation score for the mutation was 6.26 and the software-based predictions classified the variant as likely deleterious by SIFT (score 0), a disease causing by Mutation Taster (score 1.0), and probably damaging one by PolyPhen-2 (score 0.999).

A variant in the same amino acid (c. 778C>T) p. Arg260Cys position of the WNT5A protein is present in a patient with cardiovascular disease (dbSNP: rs201567461). As in the CAKUT variant the software-based predictions classified this variant as a likely deleterious one by SIFT (score 0), probably damaging as judged by PolyPhen (score 0.994) analysis. The chronic kidney disease via CAKUT is known to increase the risk for cardiovascular diseases [37]. Given this possible consequences of the *c*.*779G>A* variant to the *WNT5A* protein structure and function was studied further.

#### The *WNT5A* R260H mouse variant reduces Wnt5a signalling *in vitro*

**Fig 4:**
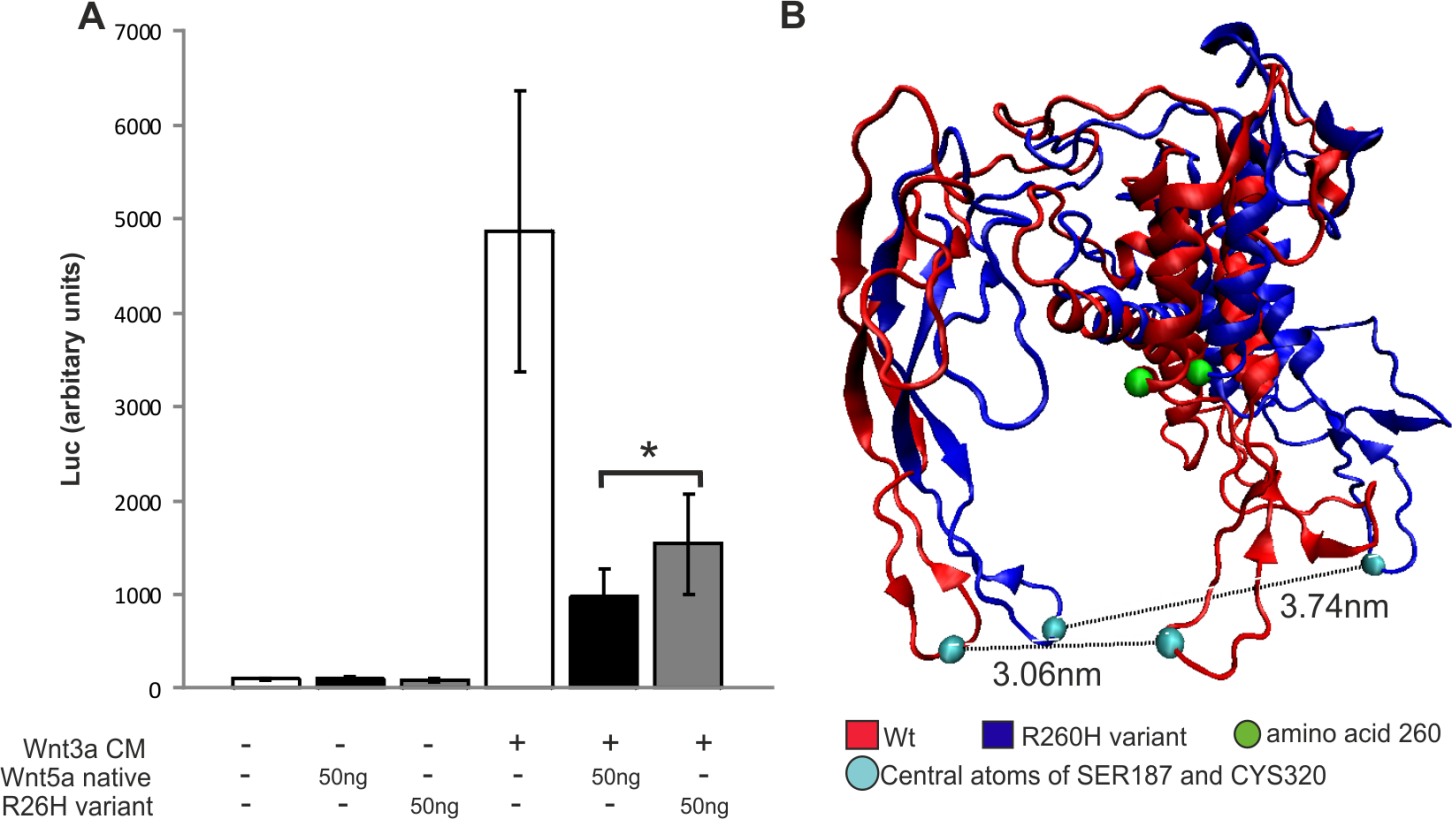
A human WNT5A R260H variant from a CAKUT cohort reduces signalling possibly via the frizzled receptor binding pocket widening as judged by simulation. The putative influence of the WNT5A R260H variant discovered in human CAKUT disease cohort was modelled by simulation by using the xWnt8 structure as the reference. A) The R260H variant in the mouse Wnt5a-GFP reduces the capacity of the Wnt5a to inhibit the Wnt3a induced *Top Flash* luciferase activity when compared to the potential of the native Wnt5a by 17% (p<0.05). B) The simulation suggests that the human R260H variant broadens the WNT5A frizzled receptor binding pocket from 3.06 nm (Wt, in red) to 3.74 nm (R260H WNT5A, in pale blue circle and dotted line). The spatial location of the residue 260 variant in the patient with CAKUT is highlighted as a green circle. CM, conditioned media. The data are shown as means ± SD, n = 7/group, * *p* < 0.05.

The influence of newly identified variant was tested directly by generating an identical mouse Wnt5a R260H variant and by inserting it to mammalian expression vector to generate transient transfection cells. The capacity of the R260H Wnt5a (R260H variant) to inhibit the canonical Wnt3a induced TOP Flash reporter served as the Wnt5a read out The results indicated that the R260H variant decreases by around 17% (p<0.05) the capacity of the Wnt5a to inhibit the Wnt3a signalling when compared to the capacity of the wild type recombinant Wnt5a transfected cells (Fig 4A).

Modelling of the putative influence of the *c*.*779G>A* to the *in silico* simulated 3D structure of the WNT5A protein suggested around 22% expansion of the frizzled receptor-binding pocked, the value changing from wild-type 3.06nm (SD 0.437nm) to the variant 3.74nm (SD 0.53nm) (Fig 4B, in red/blue, respectively). The frizzled receptor binding loop region of wild-type WNT5A protein oscillated in range of 1.5–4.3nm while the corresponding value for the R260H variant was 2.5–5.18nm. The *in silico* simulation itself does not show that receptor binding is in fact affected, the simulation result serves as a possible explanation for what was observed in *in vitro* experiments.

In summary the discovered R260H human WNT5A associated variant may have a reduced signalling capacity also *in vivo* and makes it as one putative candidate contributing risk factor for human CAKUT development.

## Discussion

Our results provide evidence that Wnt5a has a novel and specific function in mammalian kidney organogenesis. Wnt5a maintains the UB derived tree epithelial development via a mechanism that is apparently associated to secretion of the ECM components into the overlying BM. It is noteworthy that Wnt5a has a role already at the formation of the first UB branch and thereafter during UB tree development since the BM is severely affected with reduced expression of a panel of laminins and collagens at an early organogenesis stage. The BM defects lead also later to compromised kidney filter in the adult *Wnt5a* deficient mouse resembling the glomerulosclerosis condition. Based on the fact that CAKUT anomalies have their origin *in utero* as is the case with the *Wnt5a* knock out model, the impaired *WNT5A* gene may be considered a candidate CAKUT predisposing gene.

The *Wnt5a* deficiency caused kidney related phenotypes with similarity to those of *laminin γ1* characterized also by duplex collecting system or failure in organogenesis [35]. We consider this correspondence relevant for the putative mode of Wnt5a action in UB tree development. Since laminin serves as an initiator for proper BM formation [38] the noted *Wnt5a* deficiency dependent reduction of laminin and several other ECM components in the disorganized BM could be secondary reasons for primary changes in the UB cell polarity or they are direct targets of Wnt5a signalling. This possibility is supported by the presented data and those that indicated Wnt5a as being able to regulate a panel of specific BM component such *type IV collagens a1-3* and *laminins a2*, *b1-2*, *c2-3* respectively. *Wnt5a* deficiency also elevated *P4ha1* expression and the kidney glomerular BM anomalies were more pronounced in the *Wnt5a*^+/-^;*P4ha1*^*+/-*^ embryos than in the *Wnt5a* knock out only. In summary, the Wnt family member Wnt5a signalling coordinates patterning of UB development during kidney organogenesis via BM formation.

Consistent with the possible relevance in CAKUT we report here in a conserved WNT5A region a R260H (c. 779G>A, p. Arg260His) variant that was found in human cohorts with kidney diseases. As functional i*n vitro* experiments with identical mouse R260H variant showed reduction in Wnt5a capacity to inhibit Wnt3a signalling, we studied the possible explanation for the signalling reduction. *In silico* simulation of the R260H amino acid transition suggested enlargement of the frizzled receptor-binding pocket as one possible explanation. For the full proof of this hypothesis it would need to compute binding affinities for the native and the mutant with the ligand, for which structures of the ligand -bound complexes are required. Based on the acquired evidence we conclude that Wnt5a function is important for the development of the UB tree, the prospective collecting duct.

We report a specific function for Wnt5a in kidney basement membrane formation and mammalian kidney organogenesis. As a summary this provides evidence that WNT5A is a strong candidate for human CAKUT. The next step would be to conduct large-scale sequencing studies to further determine the diagnostic value of WNT5A gene and ultimately provide better support for CAKUT patients.

## Supporting Information

S1 Fig Lack of *Wnt5a* signaling causes severe anomalies in the basement membrane of embryonic kidney. Wild-type (Wt) and *Wnt5a*^-/-^ kidneys at the E16.5 were sectioned and stained with antibodies against type IV collagen (A, B) and pan-laminin (C,D). Production of type IV collagen (compare B with A, arrows) and laminin (compare D with C, arrows) in the collecting duct are reduced in the *Wnt5a*^-/-^ mice relative to Wt. Expression of certain genes encoding basement membrane components were analysed by qRT-PCR in the *Wnt5a*^-/-^ and wild-type (Wt) E16.5 kidneys (E). Expression of the genes *Lama2* (laminin α2), *Lamb1* (laminin β1), *Lamc2* (laminin γ2), *Lamc3* (laminin γ3), *Col4a1*, *Col4a2*, and *Col4a3* (α1–3 chains of collagen IV) was decreased, whereas expression of *Lamb2* (laminin β2) was increased in the *Wnt5a*^-/-^ kidneys relative to Wt. Western blotting studies depict a notable decrease in laminin-111 production in lysates derived from whole kidneys at E16.5 (G). CD, collecting duct, scale bars, A-D 100 μm.
(PDF)

S2 Fig Identification of a novel human *WNT5A* variant in a CAKUT patient. Sanger sequencing was performed for the coding region of the *WNT5A* from DNA samples that were derived from a cohort of 115 CAKUT and 14 Alport syndrome patients respectively. A) The status of the father is unknown, while the mother had been diagnosed to have CAKUT in the past. Sequence traces for the case-parent trio indicated the inheritance for the heterozygous c.779G>A variant in the patient and the father, depicted by arrows. A reference sequencing that represents a consensus of 189 healthy Dutch control individuals serves as controls. B) Alignment of the human WNT5A amino acid sequence with those of several other vertebrates reveals conservation of the amino acid sequence where the variant p. R260H transition was identified.
(PDF)

S1 Movie Visualization of the changes in development of the ureteric bud tree during *Wnt5a*^-/-^ kidney organogenesis. The ureteric bud was identified with the Troma-1 antibody at E15.5 with the aid of the optical projection tomography (OPT). A kidney of a wild-type (Wt) embryo (on the left) and the *Wnt5a*^-/-^ embryonic kidney is depicted on the right side of the OPT illustration.
(AVI)
