# Supplementary figures and images for: Wnt5a Deficiency Leads to Anomalies in Ureteric Tree Development, Tubular Epithelial Cell Organization and Basement Membrane Integrity Pointing to a Role in Kidney Collecting Duct Patterning
Source: PLoS One. 2016 Jan 21;11(1):e0147171. doi: 10.1371/journal.pone.0147171 (PMC4721645; doi:10.1371/journal.pone.0147171)

S1 Fig

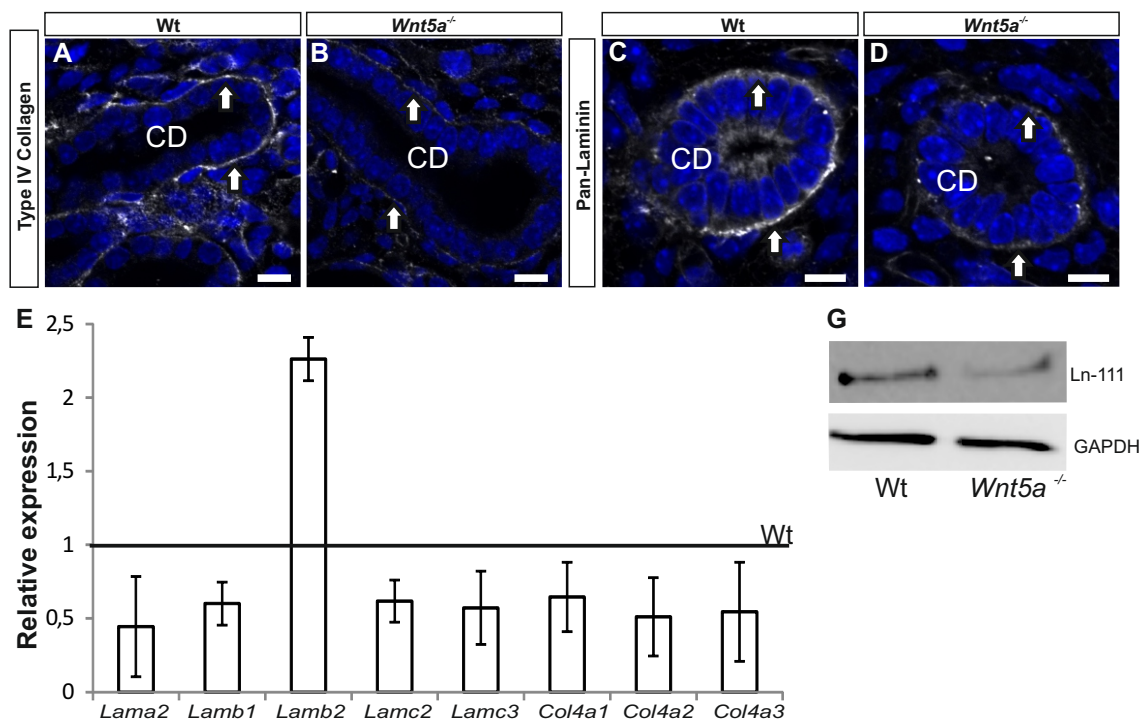

Supplement: S1 Fig — Wild-type (Wt) and Wnt5a-/- kidneys at the E16.5 were sectioned and stained with antibodies against type IV collagen (A, B) and pan-laminin (C,D). Production of type IV collagen (compare B with A, arrows) and laminin (compare D with C, arrows) in the collecting duct are reduced in the Wnt5a-/- mice relative to Wt. Expression of certain genes encoding basement membrane components were analysed by qRT-PCR in the Wnt5a-/- and wild-type (Wt) E16.5 kidneys (E). Expression of the genes Lama2 (laminin α2), Lamb1 (laminin β1), Lamc2 (laminin γ2), Lamc3 (laminin γ3), Col4a1, Col4a2, and Col4a3 (α1–3 chains of collagen IV) was decreased, whereas expression of Lamb2 (laminin β2) was increased in the Wnt5a-/- kidneys relative to Wt. Western blotting studies depict a notable decrease in laminin-111 production in lysates derived from whole kidneys at E16.5 (G). CD, collecting duct, scale bars, A-D 100 μm. (PDF) [file pone.0147171.s001.pdf]

S2 Fig

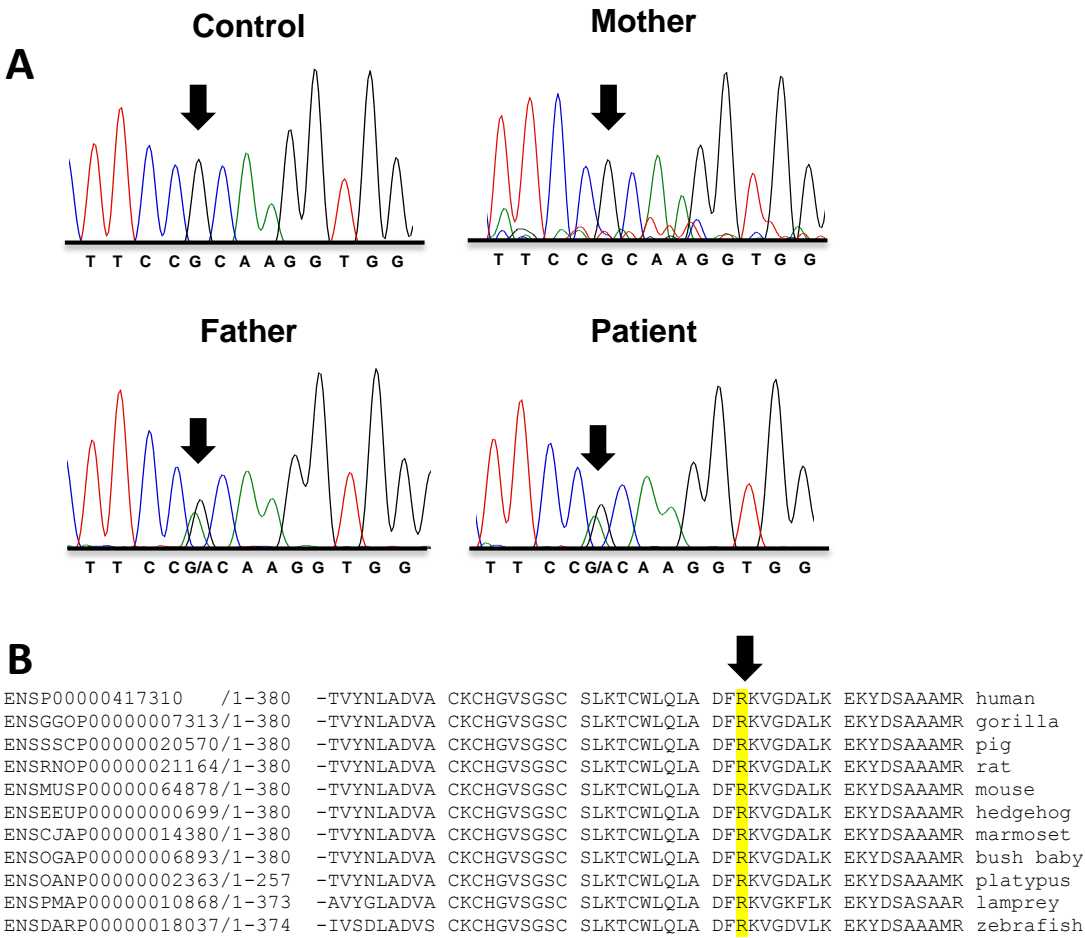

Supplement: S2 Fig — Sanger sequencing was performed for the coding region of the WNT5A from DNA samples that were derived from a cohort of 115 CAKUT and 14 Alport syndrome patients respectively. A) The status of the father is unknown, while the mother had been diagnosed to have CAKUT in the past. Sequence traces for the case-parent trio indicated the inheritance for the heterozygous c.779G>A variant in the patient and the father, depicted by arrows. A reference sequencing that represents a consensus of 189 healthy Dutch control individuals serves as controls. B) Alignment of the human WNT5A amino acid sequence with those of several other vertebrates reveals conservation of the amino acid sequence where the variant p. R260H transition was identified. (PDF) [file pone.0147171.s002.pdf]
